# RNAi-Based Screening Identifies Kinases Interfering with Dioxin-Mediated Up-Regulation of CYP1A1 Activity

**DOI:** 10.1371/journal.pone.0018261

**Published:** 2011-03-29

**Authors:** David Gilot, Nolwenn Le Meur, Fanny Giudicelli, Marc Le Vée, Dominique Lagadic-Gossmann, Nathalie Théret, Olivier Fardel

**Affiliations:** 1 EA 4427 Signalisation et Réponse aux Agents Infectieux et Chimiques, Université de Rennes 1, Institut de Recherche Santé, Environnement et Travail; Institut Fédératif de Recherche 140, Rennes, France; 2 Département Hématologie, Immunologie et Thérapie Cellulaire, Hopital Pontchaillou, CHU Rennes, Rennes, France; Instituto Nacional de Câncer, Brazil

## Abstract

**Background:**

The aryl hydrocarbon receptor (AhR) is a transcription factor activated by several environmental pollutants, such as 2,3,7,8-tetrachlorodibenzo-p-dioxin (TCDD), and involved in carcinogenesis and various physiological processes, including immune response and endocrine functions. Characterization of kinases-related AhR transduction pathway remains an important purpose.

**Results:**

We performed a kinome-wide siRNA screen in human mammary MCF-7 cells to identify non redundant protein kinases implicated in the up-regulation of cytochrome P-450 (CYP) 1A1 activity, an AhR referent target, in response to TCDD exposure. To this aim, we monitored CYP1A1-related ethoxyresorufin-*O*-deethylase (EROD) activity and quantified cell density. This normalization was crucial since it allowed us to focus only on siRNA affecting EROD activity and discard siRNA affecting cell density. Analyses of the cell density data allowed us to identify several hits already well-characterized as effectors of the cell cycle and original hits. Collectively, these data fully validated the protocol and the siRNA library. Next, 22 novel candidates were identified as kinases potentially implicated in the up-regulation of CYP1A1 in response to TCDD, without alteration of cell survival or cell proliferation. The siRNA library screen gave a limited number of hits (approximately 3%). Interestingly, four of them are able to bind calmodulin among which the IP3 kinase A (ITPKA) and pregnancy up-regulated non-ubiquitously expressed CaM kinase (PNCK, also named CaMKIβ). Remarkably, for both proteins, their kinase activity depends on the calmodulin binding. Involvement of ITPKA and PNCK in TCDD-mediated CYP1A1 up-regulation was further validated by screening-independent expression knock-down. PNCK was finally shown to regulate activation of CaMKIα, a CaMKI isoform previously reported to interplay with the AhR pathway.

**Conclusions:**

These data fully support a role for both IP3-related kinase and CaMK isoforms in the AhR signaling cascade. More generally, this study also highlights the interest of large scale loss-of-function screens for characterizing the molecular mechanism of action of environmental contaminants.

## Introduction

Halogenated aromatic hydrocarbons (AH) such as TCDD (2,3,7,8-tetrachlorodibenzo-p-dioxin) represent a major class of environmentally relevant toxicants. In mice and rats, TCDD exposure leads to a broad spectrum of adverse effects including the development of cancers and the impairment of endocrine functions and immunity. TCDD similarly exerts various major deleterious effects towards human health [1], [2], [3]. Using aryl hydrocarbon receptor (AhR) ^−/−^ mice, the pleiotropic toxicity of TCDD has been mainly linked to AhR, a basic-helix-loop-helix (bHLH) transcription factor activated after binding to cognate ligands exemplified by TCDD [4]. AhR also has important physiological roles, although the endogenous ligand(s) mediating these functions is/are as yet unidentified. For example, in AhR^−/−^ mice, vascular differentiation is severely disrupted, reproductive ability is impaired, and liver and immune abnormalities are observed [5].

After TCDD binding, AhR moves to the nucleus, dissociates from the chaperone complex, and forms a heterodimer with the AhR nuclear translocator [1]. This heterodimer binds to specific xenobiotic responsive elements found in the promoter of target genes and subsequently regulates their transcription [6]. In this way, TCDD and other AhR agonists markedly induce expression of the drug-metabolizing enzyme cytochrome P-450 (CYP) CYP1A1, which is commonly considered as a paradigm of AhR gene targets and known to detoxify and to bioactivate carcinogens [1]. AH toxicity appears to be fundamentally a consequence of dysregulation of gene expression mediated by AhR [4], [7]. Therefore the complete elucidation of AhR signaling remains an important issue.

In response to xenobiotic exposure, including TCDD and other AhR agonists, a large number of signaling pathways seems to be modulated, especially second messengers [8], [9] and protein kinases [10], [11]. However, the involvement of their modulation in regulation of target genes by xenobiotics, including that of CYP1A1 in response to TCDD, remains relatively poorly documented. These studies are recurrently based on chemical inhibition of these signaling mediators [10], although chemical inhibitors are rarely specific of only one family of kinases [12], [13]. Moreover, we and others have demonstrated that caution may be required when using chemical inhibitors to study AhR signaling since many of them are agonist/antagonist of AhR and/or inhibitor of CYP1A1 activity [14], [15], [16]. Ideally, a combination of chemical and genetic (knock-down or knock-out) inhibitions should be used to demonstrate the role of a candidate. To our knowledge, rare candidates have been identified via this double approach. Among them, members of the calmodulin-dependent protein kinase (CaMK) family are particularly interesting [17], [18], [19]. CaMKs correspond to structurally-related serine/threonine protein kinases that play important roles in proliferation [20] and differentiation [21]. We previously reported that activity of CaMKIα, one of the CaMK isoforms, is most likely required for TCDD-triggered nuclear translocation of AhR and subsequent up-regulation of AhR target genes, especially of CYP1A1 [19]. Such data therefore highlight the Ca^2+^/CaM/CaMKIα pathway as an important contributing factor to AhR function. It has also been hypothesized that other kinases, including c-src [22], CDK4 [23] and MAPKs [24], may be involved in the AhR signaling pathway.

In order to better and more extensively characterize the implication of kinases in the AhR cascade, we have analyzed the consequences of kinase expression knock-down on TCDD-mediated up-regulation of CYP1A1 activity, using a siRNA library covering the human kinome. This led us to identify 22 novel candidates as kinases potentially implicated in the up-regulation of CYP1A1 in response to TCDD, without alteration of cell survival or cell proliferation.

## Results

### A kinome RNAi-based screen allows excluding protein kinases implicated in MCF-7 cell survival and proliferation

**Figure 1:**
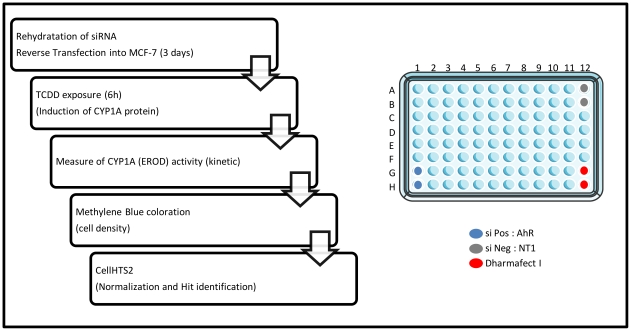
Kinome-scale siRNA screen. Flow diagram of the strategy used to identify kinases interfering with MCF-7 cell density and up-regulation of CYP1A1 activity in human TCDD-exposed cells. Negative controls (siNeg) are non-targeting siRNA (NT1)-transfected cells and Dharmafect1-exposed cells (without siRNA) (wells A12, B12 and G12, H12). Two positive controls are constituted of cells transfected with siRNA targeting AhR (siPos, G1, H1).

**Figure 2:**
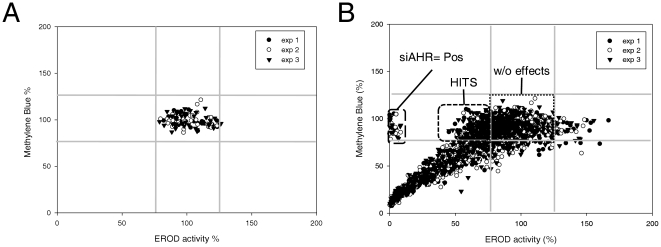
Validation of screen protocol. **A**, Scatter plot showing the global distribution of control siRNA in function of EROD activity and methylene blue data. Three experiments were shown in the same plot. For each plate, individual values of negative controls were expressed in percentage in function of the mean value of the four controls, which was set at 100%. **B**, Visualization of global distribution of all siRNA of the screen as explained for A. Negative controls: wells A12, B12 and G12, H12.

To identify nonredundant determinants of the up-regulation of CYP1A1-related ethoxyresorufin *O-*deethylase (EROD) activity in response to TCDD exposure, we designed a robust siRNA screen targeting 712 kinases of the human kinome. SiRNA were rehydrated and reverse transfected into the ERα-positive breast cancer cell line MCF-7 using Dharmafect I (Fig. 1). After 3 days, cells were exposed to 5 nM TCDD for 6 h, which usually allowed a strong induction of CYP1A1 transcription and consecutive EROD activity [19]. EROD activity was measured in kinetic in the presence of the CYP1A1 substrate ethoxyresorufin. To identify siRNA affecting cell proliferation and/or cell survival, cell density was evaluated using Methylene blue dye after the analysis of EROD activity. This normalization was crucial in our screen since it allowed us to focus only on siRNA affecting EROD activity determination and discard siRNA affecting cell density. The two sets of data (EROD activity and Methylene blue assays) were collected and analyzed using the R package cellHTS2 especially developed for cell-based high-throughput RNAi screens [25]. The methylene blue values and EROD activity values have been represented in the same plot (in x and y axis, respectively), for the negative controls (Fig. 2A) and all siRNA (Fig. 2B). These plots allowed us to visualize the global distribution of all siRNA in a same plot (Fig. 2B) and to quickly distinguish the proportion of hits. Negative controls are constituted of non-targeting siRNA (NT1)-transfected cells and Dharmafect1-exposed cells (without transfection of siRNA, (Fig. 1)). Positive controls correspond to MCF-7 cells transfected with siRNA targeting AhR, which abolished EROD activity without affecting cell density, demonstrating the efficiency of our protocol (Fig. 2B and Fig. S1). A majority of siRNA did not display any effect (Fig. 2B), as found in numerous RNAi screens [26]. A large part of siRNA affected cell survival and/or cell proliferation leading to a logical reduction of EROD activity. For the three experiments, results were quite similar (correlation factors: experiment 1 versus experiment 2: 0.79, experiment 2 versus experiment 3: 0.81 and experiment 1 versus experiment 3: 0.78, Fig. S1). It is noteworthy that each siRNA was looked at as independent of the two other siRNA targeting the same kinase, knowing that three independent screens were performed.

To further evaluate the quality of the screen and the siRNA library, a cellHTS2 analysis has been performed based on Methylene blue data (Fig. S2, Tables S1 and S2). These data have been next annotated (http://kinase.com/human/kinome/) [27], allowing the identification of 3 overrepresented kinase families (Tec, Aur and BRD) (Fig. S2C). Among these candidates, several are already known to be implicated in cell proliferation and carcinogenesis, such as Aurora kinase B and ALK [28], [29]. Interestingly, various kinases identified as regulator of cell proliferation and/or viability of MCF-7 cells can be targeted by chemical inhibitors (Fig. S2D). For example, BTK and ITK, that belong to the same Tec family, may be targeted by the LFM-A13 inhibitor [30], whereas the multikinase inhibitor AT9283 [31], a small-molecule inhibitor of kinases with potential antineoplastic activity and currently in phase II clinical trial, is able to block kinase activity of Aurora kinase B and JAK2. Altogether, these data highlighted the robustness of our RNAi-based screen approach based on loss-of-cell functions.

### RNAi-based screen identifies putative protein kinases regulating TCDD-induced EROD (CYP1A) activity in mammary MCF-7 cells

**Figure 3:**
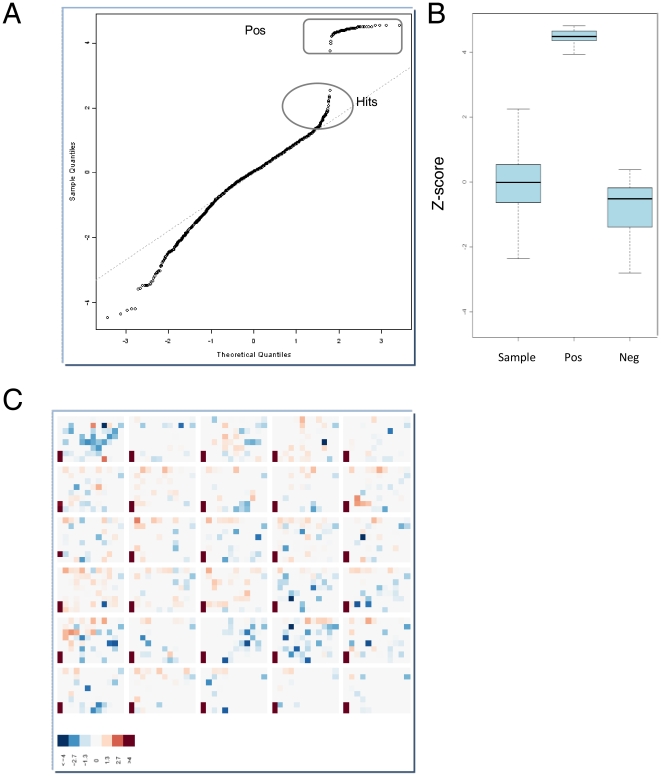
Putative kinases affecting TCDD-induced EROD (CYP1A) activity. **A**, Hit distribution on quantile-quantile plot and **B**, boxplots of z-scores for the different types of probes (sample, Pos or Neg). **C**, a screen-wide image plot to visualize the position in plates of hits. Here, the picture corresponds to z-scores calculated from 3 experiments for individual siRNA. One experiment corresponds to 30 plates. Pos for siRNA targeting AhR, Neg for negative controls constituted of non-targeting siRNA (NT1)-transfected cells and Dharmafect1-exposed cells (without siRNA) and Sample for 3×712 siRNA targeting mRNA of human kinases. Hits corresponded to the top 150 z-scores.

To identify kinases regulating TCDD-induced EROD activity, we performed a second cellHTS2 analysis using the preprocessing work-flow for two-channel screens (EROD and methylene blue data) (Fig. S3). At the end of this analysis, the module cellHTS2 displayed two complementary plots to observe hit distribution on quantile-quantile plot (Fig. 3A), and boxplots of z-scores (Fig. 3B) for the different types of probes (sample, Pos or Neg). The latest plot is a screen-wide image plot (Fig. 3C) to visualize the position of hits in plates (here, the picture corresponds to z-scores calculated from 3 experiments for individual siRNA). As illustrated in Fig. 3A, we selected the 150 siRNA causing the most significant reduction of EROD activity without affecting cell density. Next, we considered a kinase as a potential hit when at least 2 out of 3 siRNA were found into these 150 z-scores, since it is generally considered that observation of a phenotype caused by two distinct siRNA sequences indicates that it is unlikely to be the result of an off-target effect [32]. This led us to identify 22 kinases (Table 1) as candidates potentially implicated in up-regulation of EROD activity in response to TCDD, without affecting cell survival and proliferation. By contrast, with our criteria (at least 2 out of 3 siRNA), we did not identified kinases which negatively regulate the AhR pathway (that is, siRNA gene knock-down that would increase EROD activity). The siRNA library screen gave therefore a limited number of hits (approximately 3%) interfering with up-regulation of CYP1 activity.

**Table 1:** List of 22 kinases potentially implicated in the up-regulation of EROD activity in response to TCDD.

Symbol	z score (mean)	Description
ITPKA*	0.97	Inositol 1,4,5-trisphosphate 3-kinase A
PMVK*	1.26	Phosphomevalonate kinase
PNCK*	1.04	Pregnancy upregulated non-ubiquitously expressed CaM kinase
AK3	1.43	Adenylate kinase 3
BMPR2	1.49	Bone morphogenetic protein receptor, type II
CCT2	1.12	Chaperonin containing TCP1, subunit 2 (beta)
COASY	1.29	Coenzyme A synthase (COASY)
HIPK1	1.12	Homeodomain interacting protein kinase 1
IPMK	1.16	Inositol polyphosphate multikinase
LMTK2	1.08	Lemur tyrosine kinase 2
LMTK3	1.08	Lemur tyrosine kinase 3
MPP6	1.17	MAGUK p55 subfamily member 6
MYO3A	1.01	Myosin IIIA
NRK	1.12	Nik related kinase
PIK3C2G	1.00	Phosphoinositide-3-kinase, class 2, gamma polypeptide
PIM2	1.18	Pim-2 oncogene
PRPF4B	1.22	PRP4 pre-mRNA processing factor 4 homolog B (yeast)
SBK1	1.06	SH3-binding domain kinase 1
SNF1LK2	0.86	SNF1-like kinase 2
SPHK1	1.09	Sphingosine kinase 1
TRIB2	1.02	Tribbles homolog 2 (Drosophila)
UCK1	1.25	Uridine-cytidine kinase 1

The mean z-scores were calculated from three individual z-scores (3 siRNA). A kinase was considered as a potential hit when 2/3 or 3/3(*) siRNA were found into the 150 top z-scores using cellHTS2 package.

Gene ontology analysis was performed to characterize these hits (Table 2). Ten are classified as protein serine/threonine kinases and two as protein tyrosine kinases. Interestingly, four of them are able to bind calmodulin (PNCK, ITPKA, MYO3A and SPHK1). For ITPKA (IP3 kinase A) and PNCK (pregnancy up-regulated non-ubiquitously expressed CaM kinase), the kinase activity is dependent on the calmodulin binding. In addition, two hits are able to convert IP3 to IP4 (ITPKA and IPMK). The other hits (Table 1) correspond to poorly characterized kinases (i.e. Lemur tyrosine kinase 2 and 3) or non-redundant kinases AK3, PMVK, COASY, SPHK1, MYO3A. A meta-analysis was performed to integrate this list with gene expression data and protein-protein interaction data but no convincing associations were found (data not shown).

**Table 2:** Gene ontology of the 22 kinases potentially implicated in the up-regulation of EROD activity in response to TCDD.

GO	GO Coverage	Kinase	P-value
calmodulin binding	4/140 = 2%	SPHK1;PNCK;ITPKA;MYO3A	5.14E−05
calmodulin-dependent protein kinase activity	2/14 = 14%	PNCK;ITPKA	4.16E−04
inositol trisphosphate 3-kinase activity	2/7 = 28%	IPMK;ITPKA	1.08E−04
uridine kinase activity	1/4 = 25%	UCK1	9.30E−03
nucleoside triphosphate adenylate kinase activity	1/1 = 100%	AK3	3.26E−03
phosphomevalonate kinase activity	1/1 = 100%	PMVK	3.26E−03
pantetheine-phosphate adenylyltransferase activity	1/1 = 100%	COASY	3.26E−03
D-erythro-sphingosine kinase activity	1/1 = 100%	SPHK1	3.26E−03
plus-end directed microfilament motor activity	1/1 = 100%	MYO3A	3.26E−03

Annotation has been performed from http://brcabase.icr.ac.uk. P-value: right-tailed Fischer's exact test p-value adjusted for false discovery rate - the expected proportion of false discoveries amongst the rejected hypotheses. GO coverage: percentage of hits/GO genes.

### Silencing of ITPKA and PNCK decreases TCDD-induced EROD activity

Among the 22 kinases, we focused our attention on ITPKA and PNCK since they exhibited a significant impact on EROD activity whatever the siRNA sequences tested (i.e., 3 out of 3). Interestingly, we and others have already shown that calcium, calmodulin, IP3 [33], [34], and members of the CaMKs family [9], [17]–[19] actively participate to the AhR/CYP1A1 cascade.

**Figure 4:**
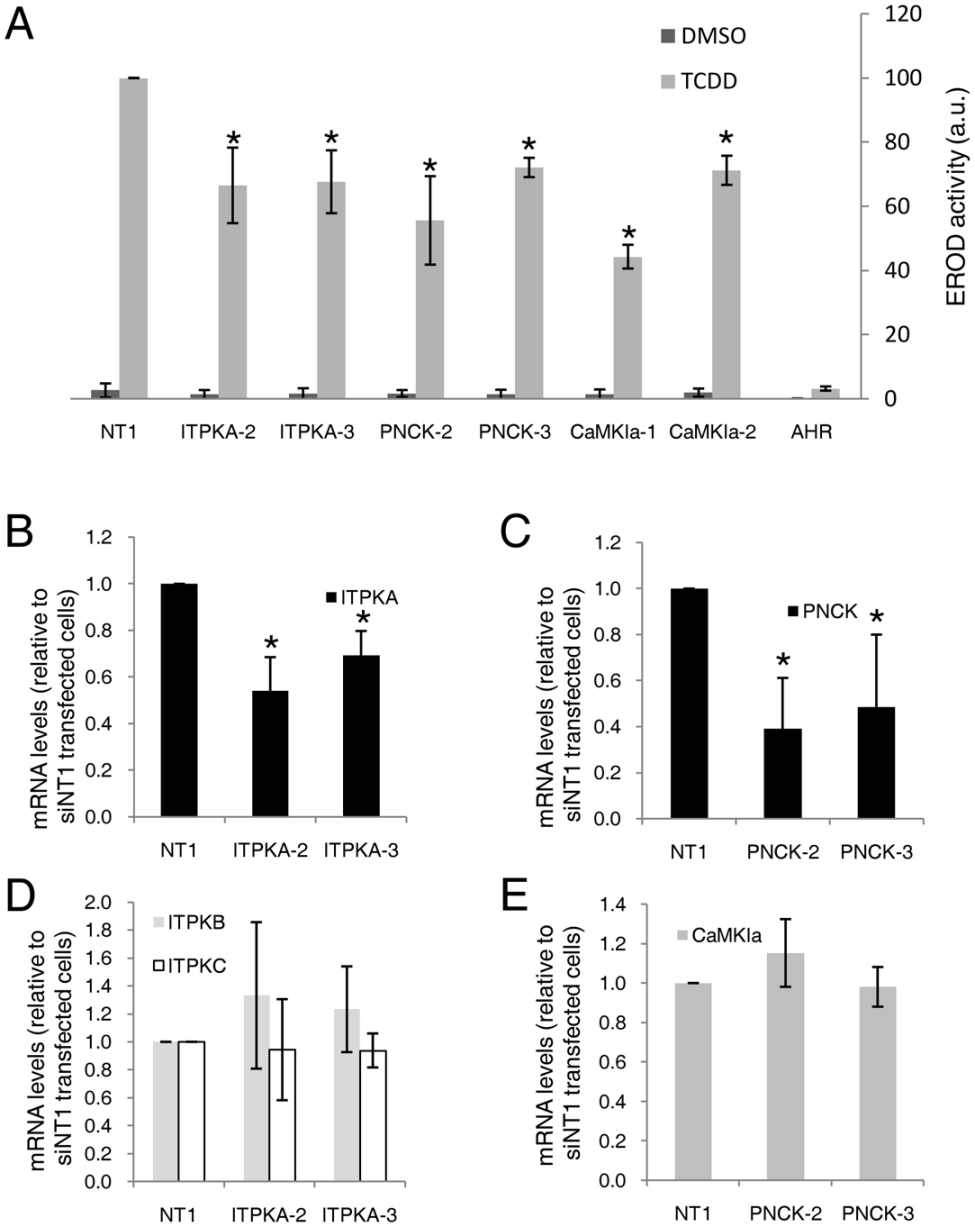
Hit confirmation and selectivity of siRNA. **A**, Knock-down of ITPKA, PNCK, CaMKIα and AhR in MCF-7 cells. In this confirmation step, 2 siRNA among 3 of the library were selected for ITPKA and PNCK and effects were compared to those of 2 siRNA targeting CaMKIα and AhR (Positive controls) and to negative control (siNT1). 72 h after transfection, cells were exposed to 5 nM TCDD for 6 h and EROD activity was measured as explained in Materials and Methods. Knock-down efficiencies were evaluated by RT-qPCR assays for ITPKA (**B**) and PNCK (**C**). Evaluation of off-target effects of siRNA targeting ITPKA or PNCK through mRNA quantification of ITPKB, ITPKC (**D**) and CaMKIα (**E**). mRNA level data are expressed in function of the values of mRNA levels found in siNT1-transfected cells exposed to TCDD, arbitrarily set to 1; they correspond to the means ± S.E.M. of three independent experiments. * p-value<0.05 compared to NT1-transfected cells.

Using two siRNA out of 3 available in our library, we confirmed that knock-down of ITPKA and PNCK expression reduced CYP1A activity in TCDD-treated MCF-7 cells. In addition, to compare the effects of ITPKA and PNCK knock-down on EROD activity to our previous published data with CaMKIα [13], [16], [19], we also used 2 other well-characterized siRNA sequence targeting this kinase [16], [19] (siCaMKIa-1 and a-2, respectively, Fig. 4A). Knock-down consequences were compared to the same controls (siNT1 and siAhR) as those used in the screen.

The knock-down of PNCK or ITPKA significantly reduced TCDD-mediated EROD activity, thus fully validating the results of the screen (Fig. 4A). SiCaMKIα-transfected cells exposed to TCDD also displayed lower EROD activity than their TCDD-treated counterparts transfected with control siNT1, in agreement with our previous data [16], [19]. Next, knock-down efficiencies and specificity of these siRNA were evaluated using RT-qPCR assays (Fig. 4B–E). Transfection of siRNA targeting ITPKA or PNCK resulted in a significant decreased expression of their targets (Fig. 4B and C). For siRNA targeting ITPKA, the mRNA expression level of the 2 other isoforms (ITPKB and ITPKC) of ITPK family was tested. ITPKA siRNAs were unable to decrease mRNA expression of these ITPK isoforms (Fig. 4D). In a similar manner, PNCK siRNAs failed to decrease mRNA expression of CaMKIα (Fig. 4E).

### PNCK governs phosphorylation state of CaMKIα on thr177

**Figure 5:**
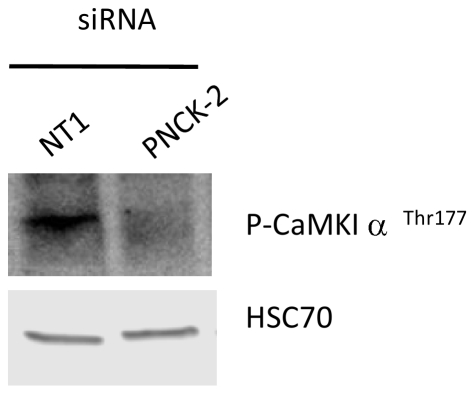
Involvement of PCNK in CaMKIα phosphorylation. MCF-7 cells were transfected with siRNA targeting PNCK or control siRNA (NT1) and 72 h later, cells were exposed to the ionophore ionomycin, a strong activator of the Ca^2+^/CaM/CaMKs pathway. Next, Western-blots were performed to analyze the phosphorylation state of CaMKIα on Thr177. Hsc70 was used as loading control. The data shown are representative of two independent experiments.

Considering the consequence of the knock-down of PNCK in the AhR/CYP1A1 pathway, we hypothesized that PNCK may be able to phosphorylate CaMKIα on Threonine 177. Such phosphorylation allows a full activation of CaMKIα as already demonstrated in rat cells [35]. MCF-7 cells were transfected with siRNA targeting PNCK or their control (NT1) and 72 h later, cells were exposed to the calcium ionophore ionomycin, a strong activator of the Ca^2+^/CaM/CaMKs cascade [36]. Next, Western-blots were performed to analyze the phosphorylation state of CaMKIα on Thr177 (Fig. 5). siRNA targeting PNCK strongly affected the phosphorylation state of CaMKIα on Thr177, thus demonstrating that PNCK is able to govern the phosphorylation state of CaMKIα on Thr177 in human cells.

## Discussion

To discover cellular factors implicated in up-regulation of CYP1A activity in response to TCDD exposure, we performed a kinome-wide siRNA screen, revealing 22 potential cellular targets that were not previously linked to this environmental pollutant. In addition, we provided the identification of a relevant set of kinases, which interfere with cell proliferation and should be consequently potential targets for breast cancer therapy.

In accordance with numerous screens using loss of cell functions [26], the number of hits down-modulating the up-regulation of CYP1A1 activity was rather limited (approximately 3%), mostly due to false negatives resulting either from the insufficient activity of some siRNA, from the high stability of some proteins, or from the redundancy of some gene functions. This RNAi-based screen failed to identify CaMKIα as interfering with TCDD-triggered EROD up-regulation. The reason for this discrepancy is rather unclear. It is not related to an alteration of cell proliferation (Table S1) and may reflect an inefficient knock-down of CaMKIα expression by the siRNA used in the screen.

In the present study we developed a more selective approach to identify truly kinase involved in EROD regulation. We first considered a kinase as a potential hit when at least 2 out of 3 siRNA were found into the 150 top z-scores (Table 1), since it is generally considered that observation of a phenotype caused by two distinct siRNA sequences indicates that it is unlikely to be the result of an off-target effect [32]. Second, we focused only on siRNA influencing the up-regulation of CYP1A1 activity in response to TCDD exposure, without concomitantly affecting cell survival and proliferation. We admitted the fact that human kinases may be necessary for these two processes. As example, the knock-down of MEK1(MAP2K1), which has been linked to AhR signaling pathway [37], strongly affected MCF-7 cell density, evaluated by methylene blue assay (Table S1). In addition, it should be kept in mind that inactivation of kinases may affect the TCDD-mediated CYP1A1 activity through an AhR-unrelated manner, such as, for example, impairment of CYP1A1 protein synthesis or catalytic activity [17], [38], [39]. Interestingly, we have not identified kinase, which negatively regulates the AhR pathway according to our criteria (at least 2 efficient siRNA out of 3) among siRNA gene knock-down that increased EROD activity (Fig. 2B).

Among the 22 kinases identified as potential hits interfering with the up-regulation of CYP1A1 activity by our RNAi screen, we confirmed the implication of PNCK and ITPKA through screen-independent transfection of efficient siRNA.

PNCK/CaMKIβ is a member of the calcium/calmodulin-dependent protein kinase family of protein serine/threonine kinases [40], known to efficiently activate CaMKIα in rat cells via Thr177 phosphorylation [35]. Interestingly, we and others previously demonstrated that Ca^2+^/CaM/CaMKIα pathway is likely an important contributing factor to AhR-mediated genomic response in MCF-7 cells, mouse hepatoma Hepa-1c1c7 cells and cortical neurons [16], [17], [18], [19], without involvement of CaMK kinases (CaMKKs), the classical upstream kinases of CaMKs [16], [19]. Intriguingly, the knock-down of only one CaMKI isoform, PNCK/CaMKIβ or CaMKIα allowed a reduction of TCDD-induced CYP1A1 activity, but not a nearly complete inhibition as obtained with siRNA targeting AhR (Fig. 4A). These effects have likely to be compared to knock-down efficiencies (siRNA targeting PNCK, ∼40–50%, CaMKIα, ∼75%, AhR, ∼85% at mRNA levels (Fig. 4C and data not shown)), and need further investigation to control the knock-down efficiencies at protein level. However, the knock-down of PNCK strongly attenuated the detection of phospho-CaMKIα on Thr177, suggesting that siRNA-mediated knock-downs are efficient for impairing functional kinase activities. This is in agreement with our previous study, in which we demonstrated that knock-down of CaMKIα abolished CaMKIα activity [19]. Finally, it is tempting to postulate that both PNCK and CaMKIα are required for Ca^2+^/CaMKI/AhR signaling pathway (see hypothetical scheme, Fig. S4). Here, PNCK was demonstrated to be able to govern the phosphorylation state of CaMKIα on Thr177 in human cells. This suggests that PNCK, unlike CaMKKs [16], [19], may activate CaMKIα cells in TCDD-treated cells.

ITPKA regulates inositol phosphate metabolism through phosphorylation of the second messenger IP3 to IP4. Interestingly, both calcium/calmodulin and protein phosphorylation mechanisms control its activity [41]. Here, transfection of siRNA targeting ITPKA decreased EROD activity in TCDD-treated cells. In addition, inositol polyphosphate multikinase (IPMK) [41], which is also able to produce IP4, has been identified as a hit. Therefore, at least two kinases converging to the same pathway were revealed as interfering with the up-regulation of CYP1A activity, suggesting a role for IP3/IP4 in the AhR/CYP1A1 pathway. Further investigations are required to elucidate the exact role of ITPKA, IPMK and of inositol polyphosphates into the AhR signaling cascade (see hypothetical scheme, Fig. S4).

In summary, our RNAi-based screen of the human kinome identifies 22 potential cellular kinases interfering with the up-regulation of CYP1A activity in response to TCDD, especially PNCK and ITPKA. These kinases were not linked to this environmental pollutant so far and could be new targets. Finally, in accordance with literature, our results showed evidence for the role of calcium, IP3, calmodulin and CaMK in the AhR pathway.

Overall, our RNAi-based screen methodology likely represents a novel opportunity to characterize signaling pathways activated by environmental pollutants.

## Materials and Methods

### Chemicals and reagents

Ethoxyresorufin and methylene blue were purchased from Sigma-Aldrich (St Louis, MO). TCDD was obtained from Cambridge Isotope Laboratories (Cambridge, MA). Ionomycin was obtained from Calbiochem (La Jolla, CA). TRIzol reagent was obtained from Life Technologies (Cergy Pontoise, France). Monoclonal mouse anti-Hsc70 and rabbit anti-phospho-CaMKIα (Thr177) Abs were purchased from Santa Cruz Biotechnology (La Perray en Yvelines, France). Chemicals were commonly used as stock solution in dimethyl sulfoxide (DMSO). Final concentration of solvent did not exceed 0.2% (v/v); control cultures received the same volume of solvent as for treated counterparts.

### Cell culture

The estrogen receptor positive cell line MCF-7 was purchased from European Collection of Cell Cultures (ECACC, Wiltshire, United Kingdom) and cultured in D-MEM medium with 4500 mg/L D-glucose, 110 mg/L sodium pyruvate and non-essential amino acids, supplemented with 100 U/ml penicillin, 100 U/ml streptomycin and 10% fetal calf serum, as previously described [19].

### Ethoxyresorufin O-deethylase (EROD) activity assay

EROD activity, corresponding to the O-deethylation of ethoxyresorufin, and mainly supported by CYP1A1 enzyme in living MCF-7 cells, was measured as previously described [19]. Briefly, MCF-7 cells were incubated in phosphate-buffered saline pH 7.4, containing 5 µM ethoxyresorufin, and kinetic reading was performed at 37°C with a SpectraMax Gemini SX spectrofluorometer over a 15 min-period.

### Methylene Blue Assay

Cell viability was assessed by a methylene blue colorimetric assay [42] immediately after EROD activity assay. Briefly, cells were fixed for at least 30 min in 95% ethanol. Following removal of ethanol, fixed cells were dried and stained for 30 min with methylene blue dye (1% in borate buffer). After two washes with tap water, 100 µL of 0.1 N HCl per well were added. Plates were next analyzed with a spectrophotometer at 620 nm.

### Reverse transfection of siRNA

The protein kinase siRNA library targeting 712 kinases of the human kinome (MISSION siRNA Human Kinase Panel, 3 siRNA per target in 3 individual wells, Table S3) has been produced and aliquoted in 96-well plates by Sigma-Genesys (St. Quentin, France). 21mer siRNA duplexes with dTdT overhangs have been produced using Rosetta Inpharmatics design algorithm. The following siRNA were purchased from Sigma-Genesys: The positive control siRNA targeting AhR (iAHR), 5′-AAGUCGGUCUCUAUGCCGCtt-3′, control siRNA (iNT1) corresponding to non-targeting siRNA, 5′-UGGUUUACAUGUCGACUAAtt-3′, siRNAs directed against CaMKIα (CaMKIα-1, 5′-GCGGUUACCCUCCCUUCUAtt-3′, and CaMKIα-2, SASI_Hs01_00226307), siRNAs directed against PNCK (PNCK-2, 5′-CCCUUUGAGGACUCGAAGAtt-3′, and PNCK-3, 5′-GCGUCUACGAGAUCCGCGAtt-3′) and siRNAs directed against ITPKA (ITPKA-2, 5′-CCUUGUGUGCUCGACUGCAtt-3′, and ITPKA-3:5′-GGCAGAAGAUCCGGACCAUtt-3′ ).

### HTS Method

Per well of 96 multiwell plate, 10 pmol of siRNAs were mixed with 0.3 µl of Dharmafect I reagent (PERBIO SCIENCES FRANCE, Brebières) diluted in 45 µl of transfection medium (Opti-MEM, Invitrogen, Paisley, UK) for 40 min at room temperature. Next, 30.000 MCF-7 cells, diluted in complete culture medium (100 µL), were added per well. 72 h later, transfected cells were exposed to 5 nM TCDD for 6 h. MCF-7 cells were next used for CYP1A-related EROD activity assay and methylene blue assay.

### CellHTS2 analysis

The dual-channel cell-based high-throughput screens (HTS) were analyzed using the R package cellHTS2 [25]. This package was especially developed to pre-process cell-based assays and is freely available on the Bioconductor project website (http://bioconductor.org/packages/2.5/bioc/html/cellHTS2.html). Briefly, data were normalized, and ratio were next calculated (ERODActivity/Methylene blue, Fig. S3). Then, data were summarized and finally z-scores were estimated, according to the preprocessing work-flow for two-channel screens, http://bioconductor.org/packages/2.5/bioc/vignettes/cellHTS2/inst/doc/twoChannels.pdf).

### RNA isolation and analysis

Total RNAs, extracted using the TRIzol method, were subjected to reverse transcription-real time quantitative polymerase chain reaction (RT-qPCR) analyses as previously described [19]. Relative quantification of mRNA levels was performed after normalization of the total amount of cDNA tested to an 18 S RNA endogenous reference. The sequences of the primers used for RT-qPCR analysis were the following: 18S sense 5′-CGCCGCTAGAGGTGAAATTC-3′, 18S antisense 5′-TTGGCAAATGCTTTCGCT-3′,

ITPKA sense 5′-GCATCGAGGGCATCAAGAAAG-3′ ITPKA antisense 5′-GATACCGCCTCAGCACTTCC3′, ITPKB sense 5′-TGGCAGGACACGCAGGGAGT-3′, ITPKB antisense 5′-GGCGAGTCGAAGTCGGCCAG-3′, ITPKC sense 5′-TGCCAGCGATCCCGAGGACA-3′, ITPKC antisense 5′-GGCTGCGCTGCTCACACTGA-3′, CaMKIα sense 5′-CAAAGATTTCATCCGGCACT-3′, CaMKIα antisense 5′-TTGAAGGCTTGCTTCACTT-3′, PNCK sense 5′-ATGACATCTCAGAATCAGCCAAAG-3′, PNCK antisense 5′-TTCCAGTGTGTCCGAGCAAAG-3′. Gene expression was calculated relative to expression of 18S endogenous control, and adjusted relative to expression in siRNA control (iNT1) transfected cells.

### Cellular protein extracts and immunoblotting analysis

Cellular protein extracts were prepared using a cell lysis buffer containing 50 mM HEPES, 150 mM NaCl, 1 mM EGTA, 0.1% Tween 20, 10% glycerol and 100 µM phenylmethylsulfonyl fluoride and supplemented with an EDTA-free cocktail protease inhibitor (Roche Diagnostic, Meylan, France). Protein samples (50 µg) were next subjected to electrophoresis in a 10% acrylamide gel and electrophoretically transferred to a nitrocellulose membrane (Bio-Rad, Marne la Coquette, France). After blocking with Tris-buffered saline containing 4% bovine serum albumin and 0.1% Tween 20 for 1 h at room temperature, membranes were incubated overnight at 4°C with primary Ab. After incubation with appropriate horseradish peroxidase-conjugated secondary Ab for 1 h, immuno-labelled proteins were visualized by autoradiography using chemiluminescence.

### Statistical analysis

Quantitative data were usually given as means ± SD of values from, at least, three independent experiments. Significant differences were routinely evaluated with the paired Student's t-test. The level of significance was p-value<0.05.

## Supporting Information

Figure S1 **Methylene blue pictures.** Pictures of 3 plates (7-9) among 30 for the 3 independent experiments. The z-scores, obtained by using cellHTS2 package for methylene blue data, were also illustrated. Negative controls: wells A12, B12 and G12, H12.
(TIF)

Figure S2 **Methylene blue data analyses.** **A**, Flow diagram of the strategy used to identify kinases interfering with cell density in MCF-7 cells. **B**, a screen-wide image plot to visualize the position in plates of hits. Here, the picture corresponds to individual z-scores calculated from 3 experiments for individual siRNA. One experiment corresponds to 30 plates. Negative controls: non-targeting siRNA (NT1) transfected cells and Dharmafect1-exposed cells (without siRNA). **C**, hits have been annotated and next classified in function of kinase families. Here, we showed only the main represented families. * indicated that at least 2 siRNA out of 3 have been identified as ‘efficient’. **D**, Eight kinases were common to our kinome-wide siRNA screen in MCF-7 cells and microarrays data obtained from 227 human breast cancers.
(TIF)

Figure S3 **R Script for the cellHTS2 analysis using the preprocessing work-flow for two-channel screens (EROD and methylene blue data).**
(TIF)

Figure S4 **Hypothetical Schema.** The diagram shows a hypothetical schema to resume the AhR signaling pathway leading to nuclear translocation of AhR and consecutive CYP1A1 up-regulation and activity (EROD). Ligands of AhR such as TCDD are known to induce a transient elevation of intracellular concentration of IP3 and calcium. Calcium and calmodulin (cam) are required for the full activation and the activity of CaMKIα, PNCK (CaMKIβ) and ITPKA. Two confirmed hits have been indicated: PNCK and ITPKA. Another hit, IPMK is also able to convert IP3 to IP4. The identity of the kinase responsible of ITPKA phosphorylation on Thr311 (symbolized by CaMK?) is probably an isoform of CaMK type II or PKA [41].
(TIF)

Table S1 **List of the individual z-scores for methylene blue data obtained from MCF-7 cells.**
(XLS)

Table S2 **List of the mean z-scores for methylene blue data obtained from MCF-7 cells.**
(XLS)

Table S3 **The human kinase library used for the RNAi screen.** Positions and sequences of siRNA.
(XLS)
