# Supplementary figures and images for: RNAi-Based Screening Identifies Kinases Interfering with Dioxin-Mediated Up-Regulation of CYP1A1 Activity
Source: PLoS One. 2011 Mar 29;6(3):e18261. doi: 10.1371/journal.pone.0018261 (PMC3066211; doi:10.1371/journal.pone.0018261)

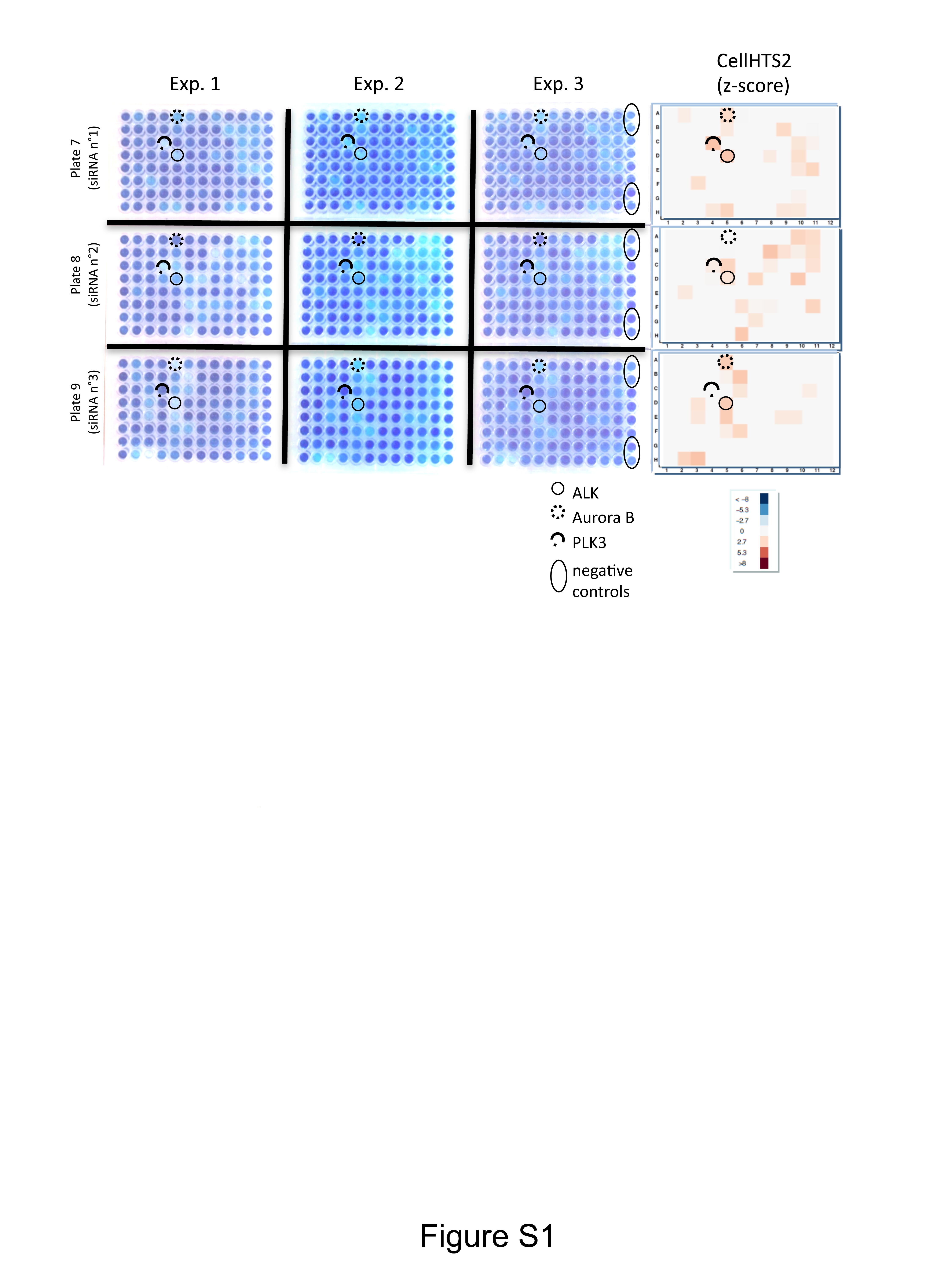

Supplement: Figure S1 — Methylene blue pictures. Pictures of 3 plates (7-9) among 30 for the 3 independent experiments. The z-scores, obtained by using cellHTS2 package for methylene blue data, were also illustrated. Negative controls: wells A12, B12 and G12, H12. (TIF) [file pone.0018261.s001.tif]

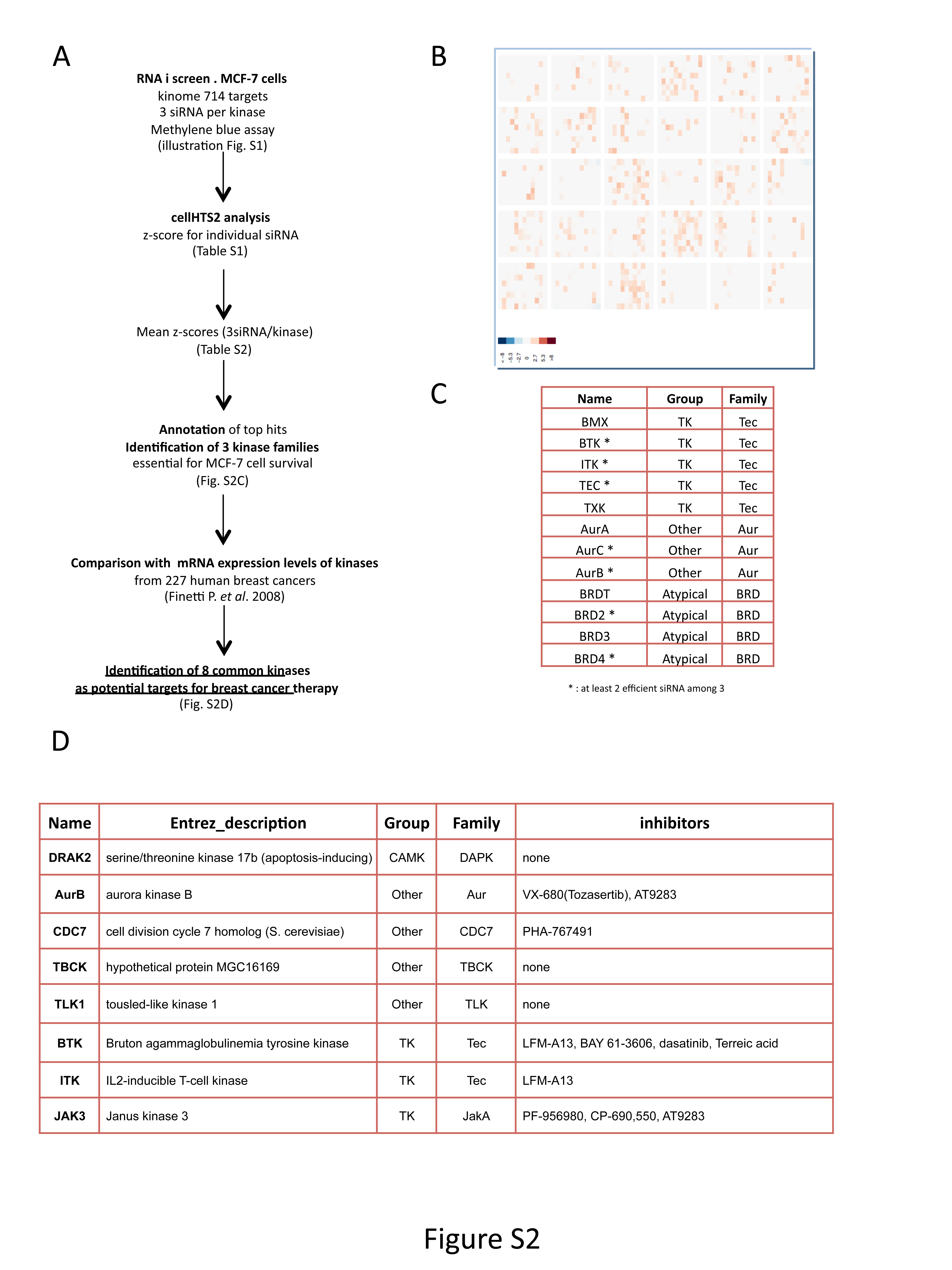

Supplement: Figure S2 — Methylene blue data analyses. A, Flow diagram of the strategy used to identify kinases interfering with cell density in MCF-7 cells. B, a screen-wide image plot to visualize the position in plates of hits. Here, the picture corresponds to individual z-scores calculated from 3 experiments for individual siRNA. One experiment corresponds to 30 plates. Negative controls: non-targeting siRNA (NT1) transfected cells and Dharmafect1-exposed cells (without siRNA). C, hits have been annotated and next classified in function of kinase families. Here, we showed only the main represented families. * indicated that at least 2 siRNA out of 3 have been identified as ‘efficient’. D, Eight kinases were common to our kinome-wide siRNA screen in MCF-7 cells and microarrays data obtained from 227 human breast cancers. (TIF) [file pone.0018261.s002.tif]

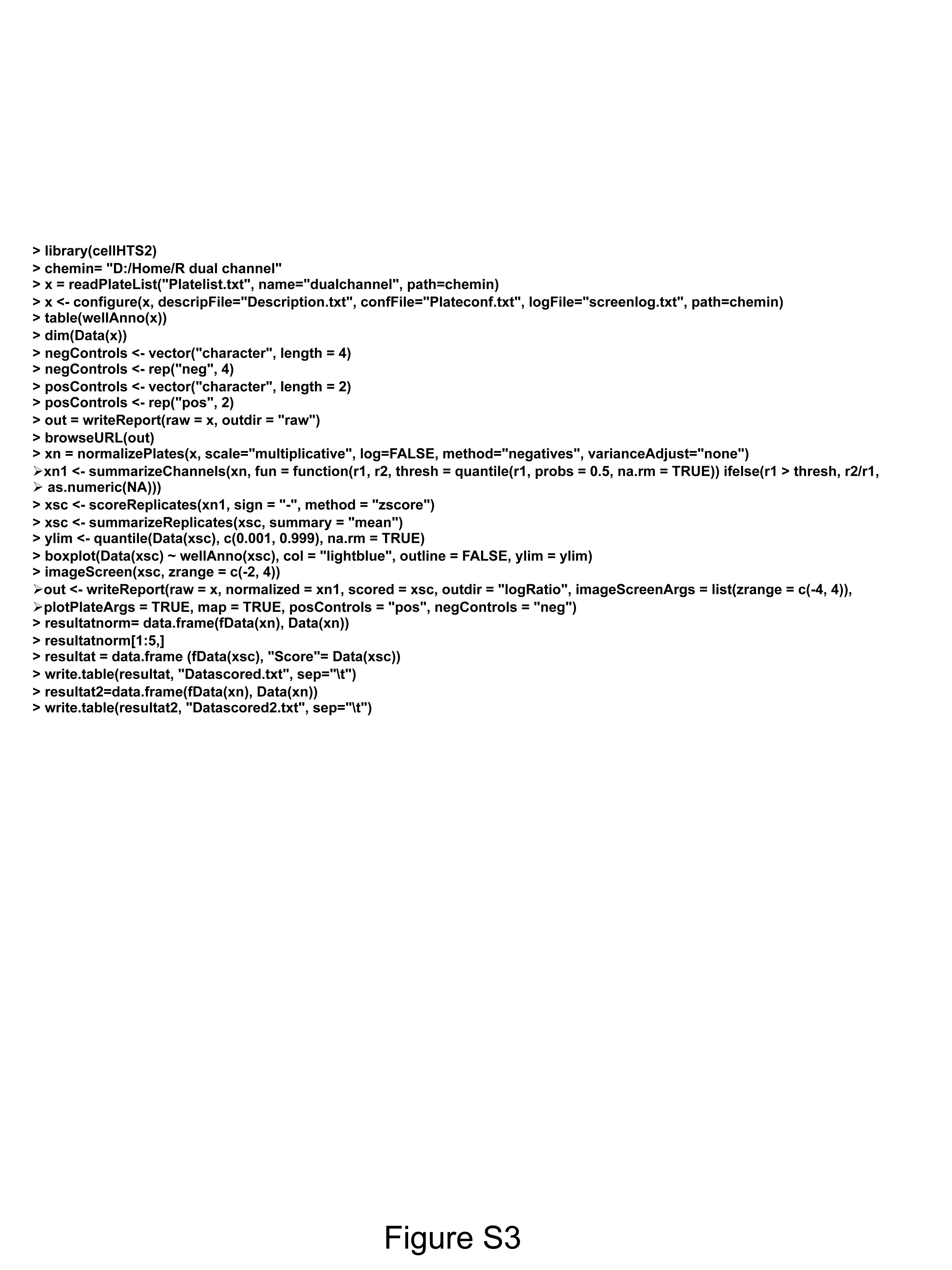

Supplement: Figure S3 — R Script for the cellHTS2 analysis using the preprocessing work-flow for two-channel screens (EROD and methylene blue data). (TIF) [file pone.0018261.s003.tif]

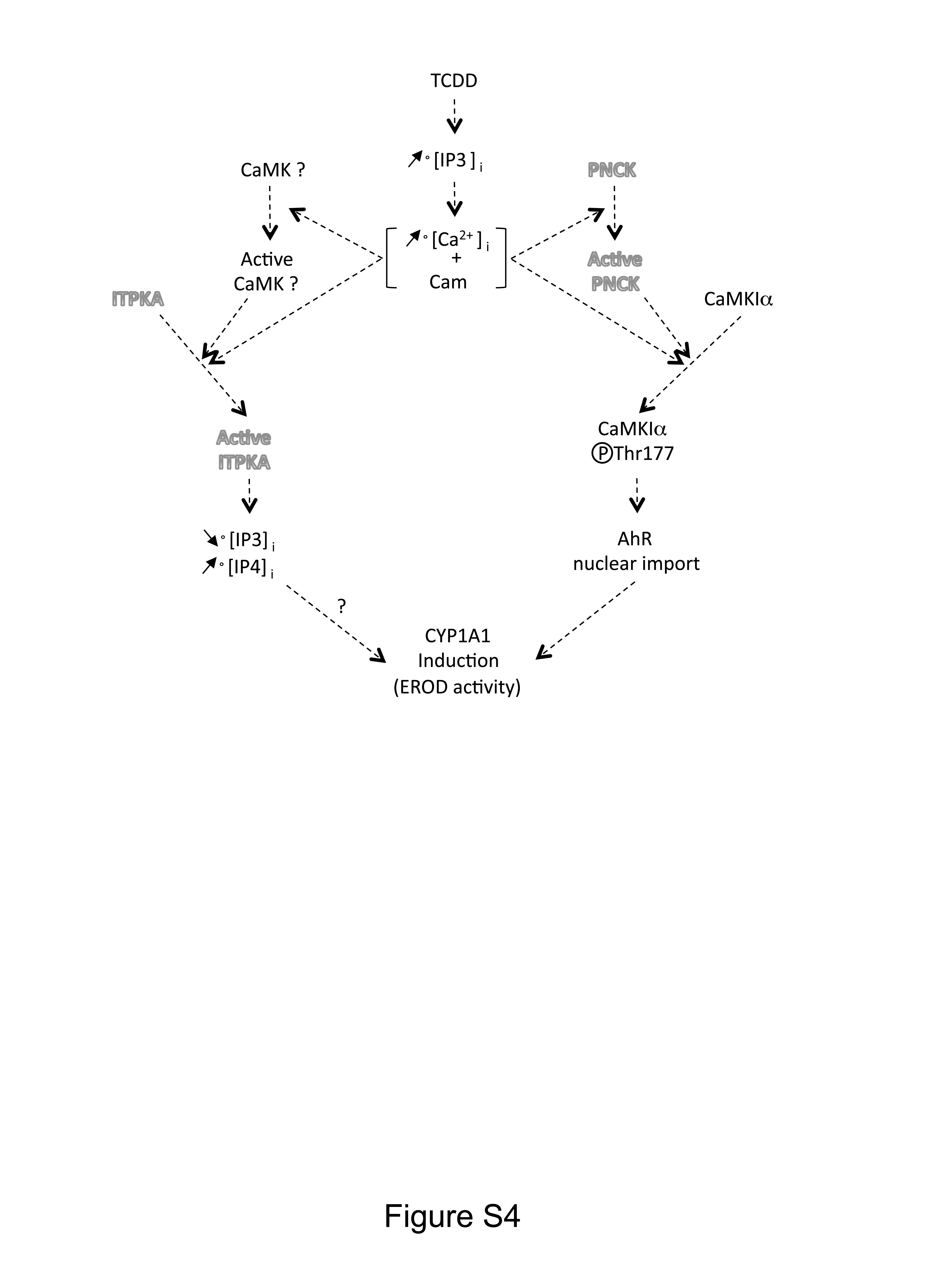

Supplement: Figure S4 — Hypothetical Schema. The diagram shows a hypothetical schema to resume the AhR signaling pathway leading to nuclear translocation of AhR and consecutive CYP1A1 up-regulation and activity (EROD). Ligands of AhR such as TCDD are known to induce a transient elevation of intracellular concentration of IP3 and calcium. Calcium and calmodulin (cam) are required for the full activation and the activity of CaMKIα, PNCK (CaMKIβ) and ITPKA. Two confirmed hits have been indicated: PNCK and ITPKA. Another hit, IPMK is also able to convert IP3 to IP4. The identity of the kinase responsible of ITPKA phosphorylation on Thr311 (symbolized by CaMK?) is probably an isoform of CaMK type II or PKA [41]. (TIF) [file pone.0018261.s004.tif]
